# Unity of Force Electricity Its Superlative Velocity

**Published:** 1877-12

**Authors:** John J. Caldwell

**Affiliations:** BALTIMORE, MD.


### ARTICLE III.

### *The Unity of Force. Electricity its Superlative Velocity.*

BY JOHN J. CALDWELL, M. D., BALTIMORE, MD.

All phenomena of the organic and inorganic world are now being interpreted in terms of matter, motion, and force. The pilot idea of the doctrine of evolution at the present day is, that everything proceeds from the simple to the complex; and that behind all the manifestations of matter and of mind, lies the "Unity of Force." It is the acting, living, persistent principle which ever sits at the loom of time, arranging the position and the texture of the threads that make up the web.

This ancient weaver, "Force," has received many appellations. When force coheres among molecules, we call it cohesive attraction. When it asserts itself among the ultimate atoms, constructing them into the delicate and beautiful crystal, we call it the crystallizing force. When engaged in uniting kindred atoms, we call it chemical force or chemical affinity. Our meaning, however, is sufficiently indicated without further extension of the list of forces.

Nature delights in prodigality of result, but she is parsimonious in the number of her working powers. Given a little matter and motion, and she will construct a world.

The lines of force radiate in all directions, always acting, always persistent. Difference of result is but the product of the same force differently conditioned. A tree and a mammal owe their origin and growth to the same parent force.

In the ultimate analysis we reach simplicity of cause and "unity of force;" there is "unity in variety and variety in unity." All that science can do, armed and equipped as she now is, if truthful to her high calling, is to recognize the important fact that, back of all working energies of nature, there lies the light of a universal intelligence, so guiding, controlling and compounding the manifold manifestations of "force," that the final outcrop of all shall redound to the best good of the creature and the everlasting glory of the Creator.

We have indicated the essential unity of "force," and the great diversity of its results. What "force" is *per se*, we know not. We can only watch the play of its untiring activities through the realm of nature.

Mysterious essence, vital "force,"
We fain would all thy secret know ;
Would find at last the hidden source
From whence thy powers ever flow.

But ours, alas! is finite sphere:
A larger vision, by and by,
May be, will solve what puzzles here,
Till then, on trusting faith rely.

Perhaps one of the most impressive instances of the play of *force* is to be seen in the phenomena of light. Light is but the result of the rapid oscillations of ether waves. Hundreds of millions of these waves strike the retina per second before we are conscious of the sensation of light. How this sensation is translated into consciousness, is a mode of force of which we know nothing. One fact, however, has been determined, viz : that color is a result of motion. Less rapid oscillations are required to produce in us the sensation of the red, in the spectrum, than of the violet. Electricity, which is the most intense of all light, is but a product of exalted motion; this can be illustrated by suspending a rod in a darkened room, with machinery attached, so as to increase its motion at pleasure. A certain degree of motion produces sound; this motion increased, produces heat; a further increase produces all the colors of the spectrum in their regular order; the utmost rapidity of motion produces electric light. What more beautiful demonstration of the unity, and yet diverse action of motion, which is only another name for "force."

But it were useless to detail a tithe of the protean results of this silent, mysterious agency. Force, being persistent, must always act; it must produce results. Harmony in results implies harmony in the conditions under which force is brought to act. Want of harmony in results implies a want of harmonious conditions. Hence what are called freaks of nature, monstrosities, and all forms of diseased action.

The theory of wave-motion in the ether had its origin in the wave-motion of the water and the atmosphere. The ear sustains the same relation to sound that the eye does to light. There is a close analogy between the harmony of sound and the harmony of colors. The rhythm of sound has its unvarying conditions in reference to the human ear. Atmospheric vibrations, when they exceed a certain number per second, lose their individuality and become merged into hum or indistinct sound. The auditory nerve is adapted to take in the whole range of the gamut. Sounds, higher or lower, in either direction, cease to be distinct. It is of no consequence whence we obtain our illustrations of the play of force; we still find it, in ultimate analysis, persistent, and a unit. The harmony of the universe is everywhere apparent. Order, design, adaptation, and law are everywhere discernible. Its workings are written in letters of living light throughout the organic and inorganic world. It flashes in the light and glows in all the colors of the spectrum. It proclaims itself in all the harmonies of sound, and in the most complex workings of the brain. Ever-boundless space is its work-shop, and never-ending time is constantly recording its mighty results.

It remains to say a word in reference to the more special manifestations of force. The human system furnishes the appropriate example. From the persistence of force we can but conclude that between the mind and the body there is the closest relation. Sensation, thought, and our most involved sentiments imply the destruction or waste of the grey tissues of the brain molecules. This waste is only another name for one mode of the play of force. In one word, man is but a product of force acting, in the first instance, on a germ, hardly microscopic. And mind with all its wonderful and complicated powers, is evolved from the simplest beginnings. A Raphæl or a Newton was evolved, in common with the lowest order of animal life, from the same dynamic, controlled and governed by the same law, which has its fitting conditions and relations. Want of harmony in these, as we have already remarked, implies want of harmony in result. A healthy mind, healthy morals, and a healthy religion, are the resultants of the unimpeded action of force.

A knowledge of the brain and of the network of the nervous system should be the aim of every physician who would minister to a body or a "mind diseased." The ability to maintain a proper electrical medium, through the exaltation or depression of nerve power, is requisite to every practitioner who would understand thoroughly, the nervous system.

Electricity's path to empire will perhaps be for a long time clogged with doubts and rejected theories. She is now making rapid strides. She is carrying the science of medicine ever onward beyond the ancient landmarks, enriching it with new truths and crowning it with new laurels.

Precisely how electricity acts as a therapeutic agent no experimenter has satisfactorily explained. Though savans, at home and abroad, have elaborated ingenious, erudite theories, still we will maintain our text, "The Unity of Force."

We will further note that electricity is but another name for the most exalted motion known to the human intellect. We are thus enabled, in some degree, to comprehend its action upon the human system, and its appropriate place in medicine, and its action through the brain and its infinite ramifications.

| | | |
|---|---|---|
| Nerve Velocity is - - | 60 | yards per second. |
| Sound " " - - | 332 | " " " |
| Cannon Ball Velocity is - | 550 | " " " |
| Light Velocity is - | 300,000,000 | " " " |
| Electricity " " - | 450,000,000 | " " " |

From the preceding table it can be readily appreciated how electricity's direct action may accelerate our natural forces, how its reverse action may depress them, or how its direct or chemical action may dissipate morbid growths or change cell structure ; how, through its instrumentality, we may dilate the vascular system, accelerate the vaso-motor nerve action, or how, through the sympathetic ganglia, exalt the trophic powers, or produce inhibitory action, which effect, as remarked by Althaus and other investigators, is caused if the galvanic current is continued, the spinal cord remains insensible to any excitation. "On breaking the circuit, mechanical or electrical excitation of the cord will again give rise to tetanic convulsions of the limbs." The pneumogastric nerve acting as an inhibitory power to regulate the heart's action, when severed upon the cardiac side, causes the heart to suddenly attain a velocity of 100 to 200 per minute.

Should the positive pole of a constant current be placed upon the proximate, and the negative upon the distal extremity of the severed nerve, with a gentle direct current in force, the inhibitory power will again manifest itself, and govern the heart's action in a normal manner. If the same experiment be tried upon the sympathetic of either side, at the cervical juncture, we have somewhat similar phenomena in regard to the nutrition of the parts; for, following the incision of the sympathetic, we have a rapid engorgement of the ear, and side of the face and neck, the parts assuming a crimson hue, from vaso-paralysis. Now, should the galvanic current be applied to the proximate and distal end, the circulation will be restored and the parts will assume their normal condition. (See experiments by Brown-Sequard, Cyon brothers, Hitzig, and others.)

Nerve velocity being but 70 to 90 yards per second, may be wonderfully increased by the influence of the direct current of electricity, especially Faradism. For, under the influence of opium, alcohol, cold, or other paralyzing agents, its velocity may be reduced until life is nearly extinct. In this lowest condition of vitality the scientific application of Faradism may restore nerve force, and re-vitalize the patient. (See my paper on "Electricity as Restorative Agent in Narcosis and Asphyxia," in the *Virginia Medical Monthly*, Nov. 1874, published by permission of the committee of the American Medical Association, 1874.)—*Dental Register.*

